# A polymer inclusion membrane composed of the binary carrier PC-88A and Versatic 10 for the selective separation and recovery of Sc

**DOI:** 10.1039/c7ra12697b

**Published:** 2018-02-26

**Authors:** Maha Sharaf, Wataru Yoshida, Fukiko Kubota, Spas D. Kolev, Masahiro Goto

**Affiliations:** Department of Applied Chemistry, Graduate School of Engineering, Kyushu University 744 Motooka Fukuoka 819-0395 Japan m-goto@mail.cstm.kyushu-u.ac.jp +81 92 802 2810 +81 92 802 2806; Center for Future Chemistry, Kyushu University 744 Motooka Fukuoka 819-0395 Japan; School of Chemistry, The University of Melbourne Victoria 3010 Australia; Centre for Aquatic Pollution Identification and Management (CAPIM), The University of Melbourne Victoria 3010 Australia

## Abstract

This study reports on the selective separation of scandium (Sc) from other rare earth metals (REMs) using a polymer inclusion membrane (PIM). The PIM prepared with PC-88A (2-ethylhexyl hydrogen-2-ethylhexylphosphonate) alone as the carrier showed high extractability but the poor back-extraction of the extracted Sc^3+^ ions did not allow the transport of these ions to the receiving solution of a membrane transport system. To overcome this problem, a novel approach was introduced using a mixture of carriers that allowed Sc^3+^ transport into the receiving solution. A cellulose triacetate (CTA) based PIM containing both PC-88A and Versatic 10 (decanoic acid) as carriers and dioctyl phthalate (DOP) as a plasticizer was prepared for the selective separation of Sc^3+^ from other REM ions in nitrate media. The membrane composition was optimized and the effect of operational parameters such as pH of the feed solution and composition of the receiving solution was explored. The flux at the membrane/feed solution interface was found to depend significantly on the carrier concentration in the PIM, pH of the feed solution and the receiving solution acidity. The newly developed PIM allowed quantitative and selective transport of Sc^3+^ thus demonstrating its suitability for the selective recovery of this metal.

## Abstract 2

This study reports on the selective separation of scandium (Sc) from other rare earth metals (REMs) using a polymer inclusion membrane (PIM).

## Graphical abstract

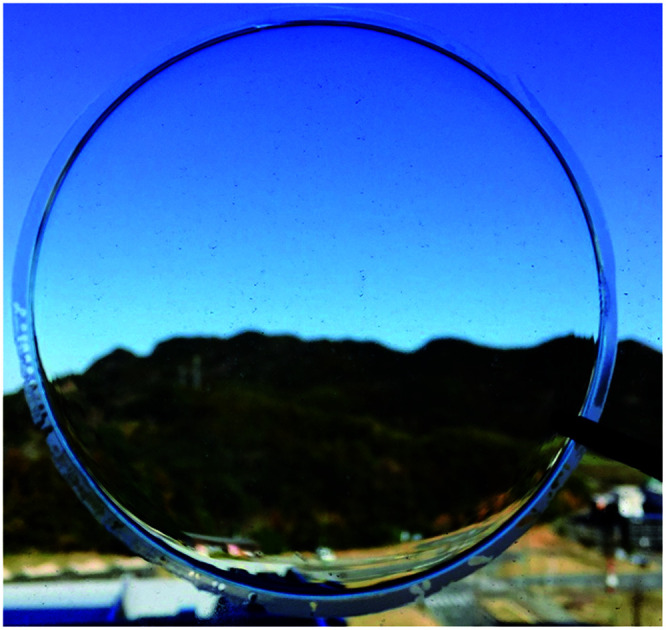

## 1 Introduction

The demand for rare earth metals (REMs) has been steadily increasing due to their unique physical and chemical characteristics and extensive usage in various life sectors, *e.g.*, adding a small amount of REMs to steel can improve its heat, corrosion and oxidation resistance.^1^ Scandium is a REM, which has an essential role in many application areas such as aluminium alloys, semiconductors and fuel-cells.^2^ The price of scandium is considerable and it is produced mainly as a by-product, therefore, there is an urgent need for the development of dedicated and efficient separation techniques to sustain the growing demand for this metal. Solvent extraction is one of the industrially established separation methods to recover REMs from aqueous solutions.^3,4^ However, it still has some significant drawbacks such as requiring relatively complex equipment, consuming high amounts of solvents, involving multi-stage extraction processes, extensive energy consumption, and limited ligand selection.^5^

In recent years, liquid membrane technologies have attracted much attention in the separation and recovery of metal ions from aqueous solutions^6^ because of their high energy efficiency, flexibility, low cost, easily satisfying environmental pollution regulations, and offering controlled membrane permeability. There are several kinds of liquid membranes for metal separation from aqueous liquors, such as supported liquid membranes (SLMs), emulsion liquid membranes (ELMs), bulk liquid membranes (BLMs), and polymer inclusion membranes (PIMs).^7^ In the present study, we have focused on PIMs due to several important advantages they offer compared to the other types of membrane mentioned above.

PIMs are liquid membranes which consist of a base polymer (usually poly(vinyl chloride), PVC or cellulose triacetate, CTA), a carrier (often a commercial solvent extractant) which is immobilized between the entangled chains of the base polymer, and a plasticizer, if necessary.^8–10^ PIM-based separation is considered as a highly promising technique because of the high stability, selectivity, efficiency, and durability of PIMs.^11^ The outstanding performance of PIMs in comparison with the other types of liquid membranes is expected to lead to the development of novel industrial separation processes in the near future. PIMs have already been shown to be applicable to the separation of some REMs,^12–14^ actinides^15,16^ as well as other metals.^17–24^ However, there have been no reports so far on scandium separation using PIMs because of the lack of suitable extractants/carriers. There are several good extractants with high extraction affinity for scandium,^25^ however, back-extraction is known to be difficult when such extractants are used. To establish a successful membrane separation system, the efficiency of back-extraction is equally important as that of extraction.

In our previous solvent extraction study,^26^ we found that a binary extractant composed of PC-88A and Versatic 10 showed a good performance for both the selective extraction and efficient back-extraction of scandium. The present paper reports on the development, optimization and characterization of the first PIM for the selective separation of the scandium ion from other REM ions.

## 2 Experimental

### 2.1 Reagents and chemicals

PC-88A and Versatic 10 were supplied by Daihachi Chemical (Osaka, Japan) and Japan Epoxy Resin (Tokyo, Japan) (currently Mitsubishi Chemical Corporation), and used without further purification. CTA used as the PIM base-polymer, dioctylphthalate (DOP) and 2-nitrophenyloctyl ether (2NPOE) used as plasticizers were purchased from Sigma-Aldrich (St Louis, USA). The salts of the REMs studied were provided by Aldrich and used in the preparation of the feed solutions. Sulfuric acid, nitric acid and ammonium nitrate were purchased from Kishida Chemical (Osaka, Japan).

### 2.2 Membrane preparation

**Fig. 1:**
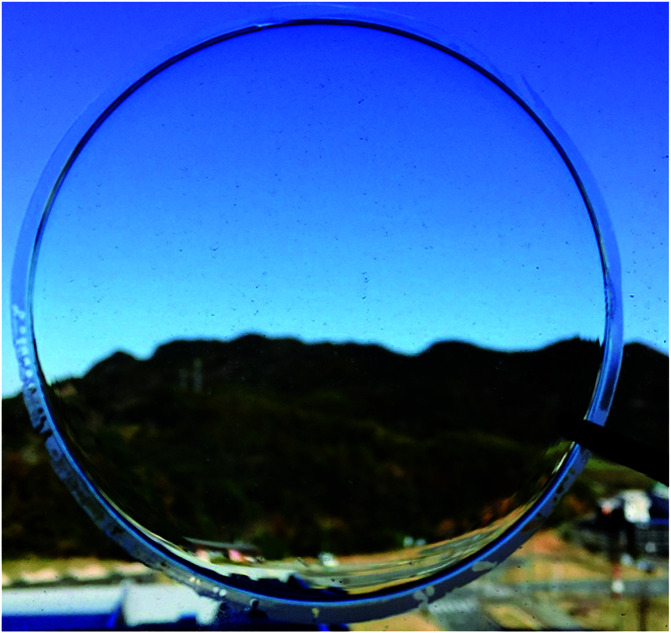
A photo image of a prepared membrane.

In this study, PIMs using CTA as the base-polymer, and dioctylphthalate (DOP) and 2-nitrophenyloctyl ether (2NOPE), used as plasticizers were prepared as previously described.^27^ A homogeneous solution containing the required amounts of CTA (20–70 wt%), the plasticizer (0–40 wt%) and the mixed carrier (0–40 wt%) with a total mass of 400 mg was prepared in 10 mL dichloromethane. The ratio of PC-88A and Versatic 10 was also varied. The solution was then poured into a 7.5 cm in diameter glass ring positioned on a flat glass plate, and covered with a filter paper and a watch glass to allow the slow evaporation of the solvent overnight. The prepared membranes were transparent with a soft surface and a good mechanical strength as shown in Fig. 1. Membrane thickness was measured using Digimatic Micrometer MDC-25 MX (Mitutoyo, Japan) and the corresponding value was calculated as the average of 10 spots along the membrane diameter. The average thickness of the membranes used in all experiments was 60 ± 5 μm.

### 2.3 Membrane extraction and back-extraction experiments

The PIM extraction experiments were conducted by immersing a circular segment of the membrane with an average mass of 80 mg in a 50 mL of an aqueous feed solution in a glass flask covered with Parafilm. To evaluate the effect of parameters on the PIM performance, all experiments were performed at a constant pH of 4, unless otherwise stated. All extraction experiments were performed at a stirring rate of 90 rpm at 25 °C using a thermostated orbital shaker (EYELA NTS-4000, Tokyo Rikakikai Co., Ltd., Tokyo, Japan). The aqueous feed solutions were prepared by dissolving metal salts in mixtures of 0.1 M HNO_3_ and 0.1 M NH_4_NO_3_ solutions. The pH of the feed solutions was adjusted by varying the ratio of the HNO_3_ and NH_4_NO_3_ solutions. The pH during the extraction experiments was measured at predetermined times by using a pH meter (H-M-60 G, DKK-TOA, Japan). Samples (1 mL) were taken periodically from the feed solution and replaced with the same volume of the initial feed solution. Metal concentrations were analyzed by inductively coupled plasma-atomic emission spectrometry (ICP-AES, Optima 8300; Perkin Elmer, USA).

The kinetics of the PIM extraction was described by the following first order equation:1
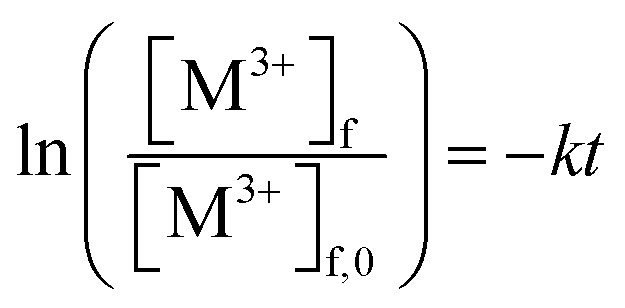
where *t* is the time (h), [M^3+^]_f,0_ and [M^3+^]_f_ are the concentrations of the REM ion in the feed solution at times 0 (original solution) and *t*, respectively, and *k* is the kinetic rate constant (h^−1^) for the metal uptake into the membrane.

The back-extraction experiments were conducted by immersing the loaded with a REM ion membrane into a sulfuric acid receiving solution.

### 2.4 Membrane transport experiment

**Fig. 2:**
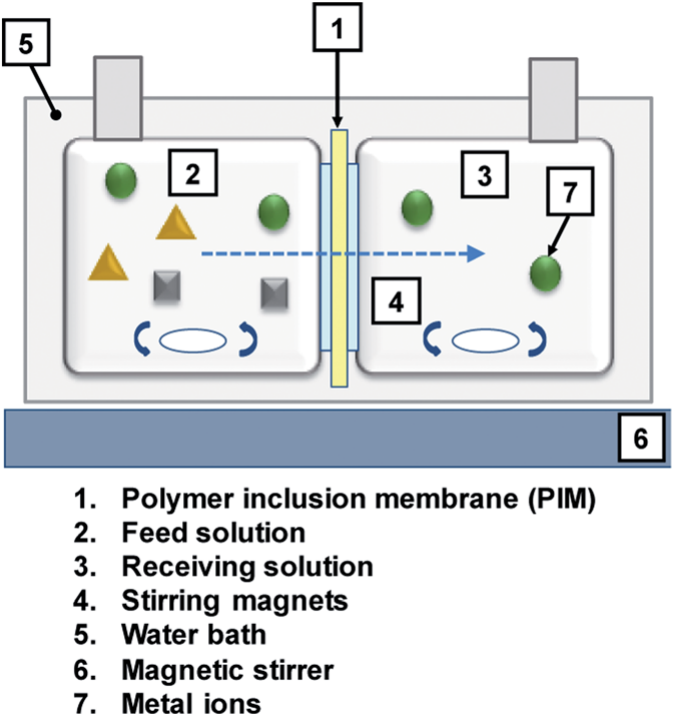
Schematic diagram of the PIM transport cell.

Metal transport experiments were performed using a transport system consisting of two jacketed glass compartments with the PIM sandwiched between them as shown in Fig. 2. The membrane apparatus is designed for analyzing a transport behavior, and the same apparatus was used in the study of supported liquid membranes (SLMs) experiments.^28,29^ The two compartments were filled with the feed and receiving solutions (50 mL each). The diameter of the membrane exposed to the solutions was 25.0 mm (effective surface area of 4.9 × 10^−4^ m^2^). The solutions in both compartments were mechanically stirred at 300 rpm during the experiments using magnetic stirrers (KH-55D, Vidrex, Japan) and small crosshead magnetic stirring bars (8 × *ϕ*10). Samples (1 mL) were taken from the ports of both glass compartments at predetermined times and analyzed by ICP-AES. The samples were replaced with the same volume of the corresponding fresh solutions. Temperature of both compartments was kept constant at 25 °C by continuously circulating water through the compartments' jackets from a water bath with a thermoregulator (RCB-1200, EYELA, Japan). The feed solution containing initially 0.1 mM metal ion(s) and the receiving solution were prepared as described earlier. As in the extraction experiments, it was assumed that the transport kinetics was first order (eqn (1)). The permeability coefficient (*P*), initial flux *J*_0_ (mol m^−2^ s^−1^) and recovery factor RF (%) were determined by eqn (2)–(4), respectively.2
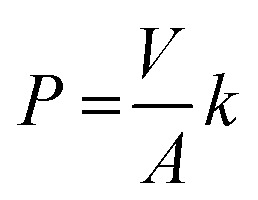

3 *J*_0_ = *P*[M^3+^]^f^_0_

4
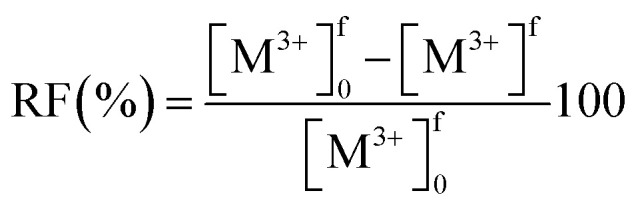
where *V* (m^3^) is the volume of feed solution, *A* (m^2^) is the membrane surface area exposed to each of the two solutions, and superscript f refers to the feed solution.

### 2.5 Membrane characterization

The morphology of the PIMs studied was examined by imaging their cross-sections using scanning electron microscopy (SEM, Inspect S50 XT microscope, type: FB 2017/12-FEI, USA). The membrane samples were first immersed into liquid nitrogen for 15 min, then were cut into small rectangular pieces to be fixed onto the sample holder, and finally were coated with palladium (MSP-15 Magnetron Sputter Hitachi, Japan). SEM images at two different scales were taken.

## 3 Results and discussion

### 3.1 Optimization of the PIM composition

The optimization of the composition of the CTA-based PIM containing PC-88A and Versatic 10 was based on extraction and back-extraction experiments involving PIMs with different compositions.

#### 3.1.1 Carrier

**Fig. 3:**
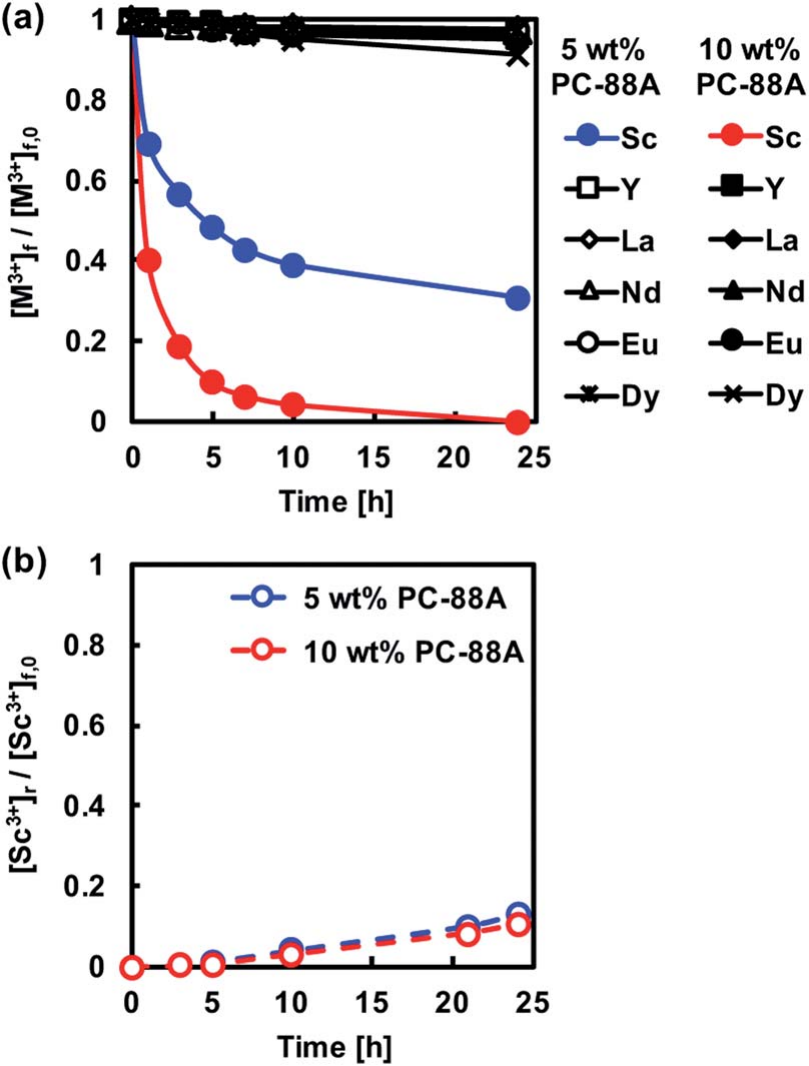
(a) Normalized transient concentrations in the feed solution of the REM ions studied during the PIM extraction experiments. (b) Normalized transient Sc^3+^ concentration in the 1 M sulfuric acid receiving solution during the PIM back-extraction experiments. Experimental conditions: CTA-based PIMs composition – 5 or 10 wt% PC-88A and 30 wt% DOP; PIM mass and thickness – 80 mg and 60 ± 5 μm, respectively; feed solution volume, initial composition and pH – 50 mL, 0.1 mM of each of the REM ions studied and 1, respectively.

Initially, PIMs containing either PC-88A or Versatic 10 were studied. Fig. 3(a) shows the transient concentrations of REM ions ([M^3+^]) of interest in the feed solution normalized with respect to their initial concentrations ([M^3+^]_0_) during extraction experiments at pH 1 involving PIMs containing different concentrations of the extractant PC-88A and 30% of the plasticizer DOP. The results showed that Sc^3+^ was selectively extracted into the membranes and the extraction rate and amount extracted increased with increasing the PC-88A concentration. Upon increasing PC-88A concentration in the PIM up to 10%, the extracted amount of Sc^3+^ gradually increased and quantitative extraction of Sc^3+^ into a PIM containing 10% PC-88A was achieved after 24 h of extraction. In experiments with feed solutions at pH 4 other REMs were also extracted and this lowered the extraction rate and the amount of Sc^3+^ extracted. However, it was difficult to back-extract Sc^3+^ from the PC-88A containing PIMs regardless of the concentration of PC-88A as shown in Fig. 3(b) where the transient concentration of Sc^3+^ is normalized with respect to the initial concentration in the feed solution ([Sc^3+^]_0_). The inability to back-extract quantitatively Sc^3+^ from the PIM would be a serious obstacle in developing a PIM-based separation technology for this metal.

**Fig. 4:**
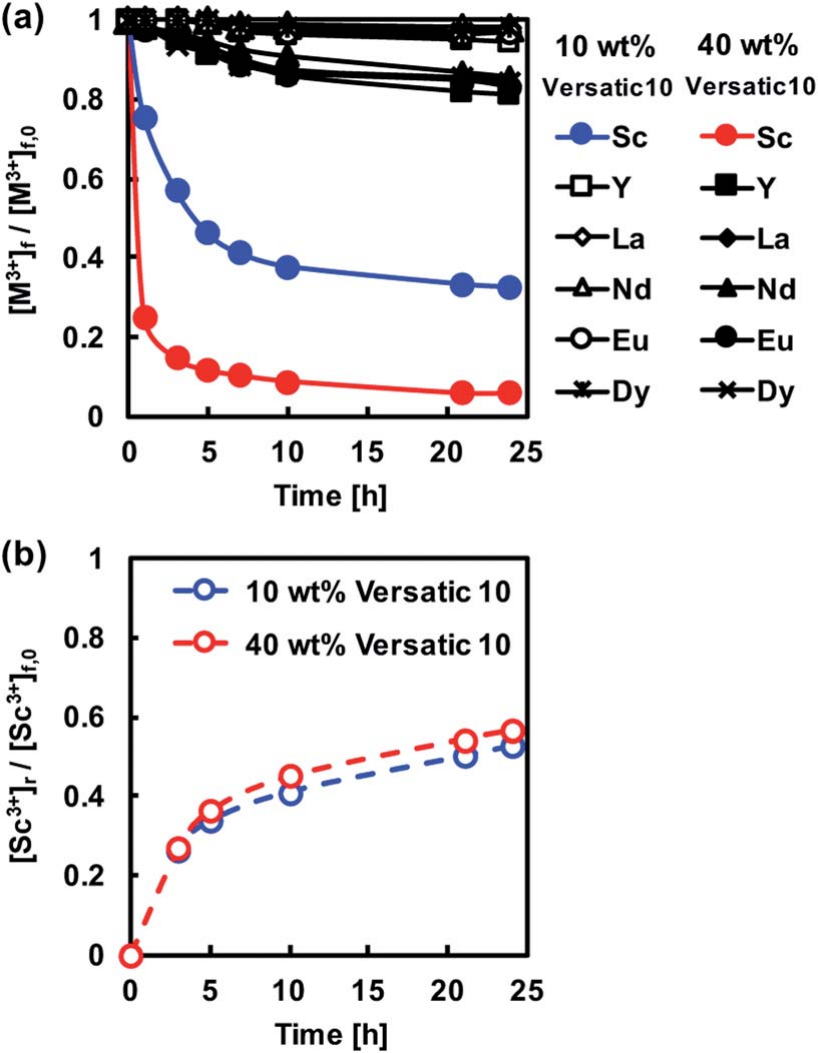
(a) Normalized transient concentrations in the feed solution of the REM ions studied during the PIM extraction experiments. (b) Normalized transient Sc^3+^ concentration in the 1 M sulfuric acid receiving solution during the PIM back-extraction experiments. Experimental conditions: CTA-based PIMs composition – 10 or 40 wt% Versatic 10 and 30 wt% DOP; PIM mass and thickness – 80 mg and 60 ± 5 μm, respectively; feed solution volume, initial composition and pH – 50 mL, 0.1 mM of each of the REM ions studied, 4, respectively.

The membrane extraction of REM ions was also carried out using PIMs with Versatic 10 alone as the carrier. The extraction ability of the extractant Versatic 10 for Sc^3+^ is considerably lower than that of PC-88A^25^ and therefore the extraction experiments involving Versatic 10-based PIMs were conducted at feed solution pH of 4 rather than 1 (Fig. 4(a)). As expected, the back-extraction of Sc^3+^ from the Versatic 10-based PIMs was found to be better than that from the PC-88A-based membranes (Fig. 4(b)).

It has been proven that the use of binary extractants in solvent extraction systems^30^ can improve their ability to selectively and reversibly extract specific target metal ions. In our previous study, we have found that the binary extractants PC-88A and Versatic 10 are suitable for both the selective extraction and efficient back-extraction of Sc^3+^.^26^ It was confirmed that Sc^3+^ is extracted *via* the exchange by three protons in the binary extractant system. The reaction seemed to involve predominantly PC-88A, and Versatic 10 was considered to reduce the affinity of PC-88A to Sc^3+^. This result improved the back-extraction efficiency. Therefore, the possibility of using this binary extractant as the carrier of a CTA-based PIM was examined as a part of the present study. It was expected that the presence of Versatic 10 would improve the stripping efficiency of Sc^3+^ from the PIM with the binary extractant compared to the PIM containing only PC-88A while keeping the high selectivity of the latter membrane.

**Fig. 5:**
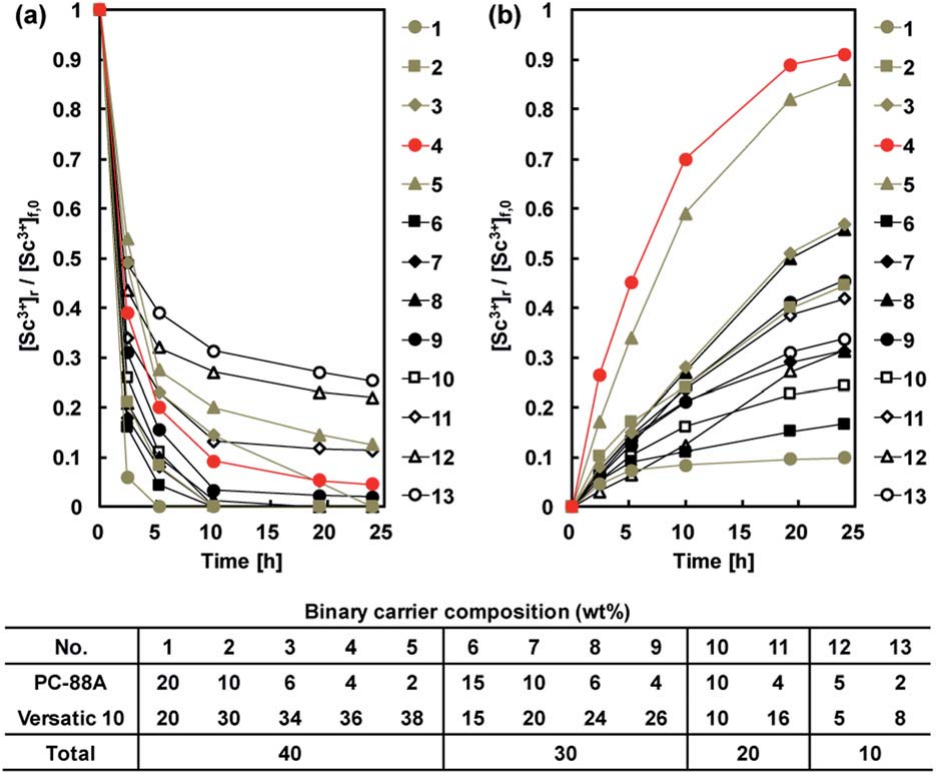
(a) Normalized transient Sc^3+^ concentration in the feed solution during the PIM extraction experiments. (b) Normalized transient Sc^3+^ concentration in the 1 M sulfuric acid receiving solution during the PIM back-extraction experiments. Experimental conditions: CTA-based PIMs composition – binary extractant (composition in the above table) and 30 wt% DOP. Remaining experimental conditions as in Fig. 4.

The extraction and back-extraction characteristics of CTA-based PIMs containing 30 wt% DOP as plasticizer and both PC-88A and Versatic 10 as the binary carrier in concentrations equal to 10 wt%, 20 wt%, 30 wt%, and 40 wt% were studied. The ratio between the two extractants was varied, while all the extraction experiments were conducted with the same initial feed solution concentration of 0.1 mM and pH of 4. Best extraction was achieved at this pH value in a series of experiments where the initial pH was varied between 1 and 4. It should be noted that in the course of the extraction process the pH decreased from 4 to around 3.3. As shown in Fig. 5, the extraction of Sc^3+^ was enhanced by increasing the PC-88A concentration of the membrane, however, the back-extraction of Sc^3+^ was depressed as a result of this. Moreover, increasing the Versatic 10 percentage over that of PC-88A was found to be effective for improving the back-extraction efficiency. Both quantitative extraction and back-extraction were achieved with a PIM containing 40 wt% carrier (4 wt% PC-88A + 36 wt% Versatic 10), 30 wt% CTA and 30 wt% DOP. PIM with concentration of the carrier greater than 40 wt%, was found to be mechanically unstable and therefore the combination of 4 wt% PC-88A and 36 wt% Versatic 10 was selected as the optimum carrier composition. The results mentioned above demonstrated the importance of controlling the ratio between the two extractants for achieving efficient extraction and back-extraction of Sc^3+^. The initial rate constant (*k*) values calculated by eqn (1) for different carrier compositions in the membrane are summarized in Table 1.

**Table 1:** Rate constant (*k*) values for Sc^3+^ extraction in PIMs of different carrier composition. Remaining experimental conditions as in Fig. 4

Carrier compositions	DOP (wt%)	*k* [h^−1^]
10 wt% PC-88A*	30	0.29
40% Versatic 10	30	0.36
4 wt% PC-88A + 36 wt% Versatic 10	30	0.55
4 wt% PC-88A + 36 wt% Versatic 10	40	0.86

#### 3.1.2 Plasticizer

**Fig. 6:**
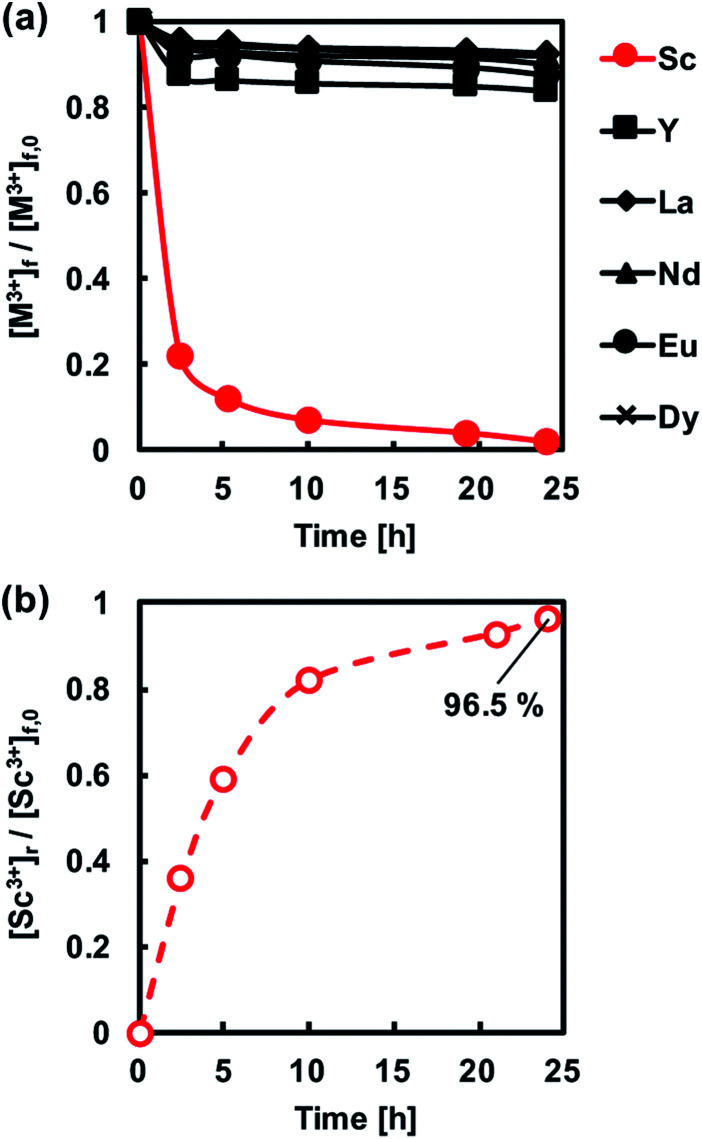
(a) Normalized transient concentrations in the feed solution of the REM ions studied during the PIM (4 wt% PC-88A and 36 wt% Versatic 10) extraction experiments. (b) Normalized transient Sc^3+^ concentration in the 1 M sulfuric acid receiving solution during the PIM back-extraction experiments. Remaining experimental conditions as in Fig. 4 except for the DOP concentration which was 40 wt%.

CTA-based PIMs containing 4 wt% PC-88A and 36 wt% Versatic 10 as the binary extractant and DOP or 2NPOE as the plasticizer in the concentration range of 0–40 wt% were studied. The experimental results suggested that the membrane extraction rate increased upon increasing the percentage of the plasticizer in the membrane (data not shown). The best extraction and back-extraction efficiency was achieved when using 40 wt% DOP. The extraction and back-extraction results obtained at this PIM composition are shown in Fig. 6, and the initial extraction rate constant values are listed in Table 1. It can be expected that by increasing the plasticizer concentration, the viscosity of the membrane liquid solution will decrease thus providing more opened polymer structure and lower membrane diffusion coefficients.^31^ The PIMs prepared with 2NPOE as the plasticizer were less flexible than those containing DOP. In addition, DOP is the less expensive plasticizer. Therefore, DOP was considered to be the more suitable plasticizer for preparing the PIMs with optimal composition.

#### 3.1.3 Stripping reagent

Three different mineral acids (HCl, HNO_3_ and H_2_SO_4_) were studied for their suitability as stripping reagents for Sc^3+^ and it was established that their stripping efficiency increased in the order HNO_3_ > HCl > H_2_SO_4_. However, when we used the nitric and hydrochloric acids as the receiving solution in membrane transport experiments, the pH values in the feed solution drastically dropped down. Therefore, in the membrane transport experiments, sulfuric acid was selected due to its high hydrophilicity, which protected the acid transport from the receiving solution to the feed one.

### 3.2 Membrane characterization by SEM

**Fig. 7:**
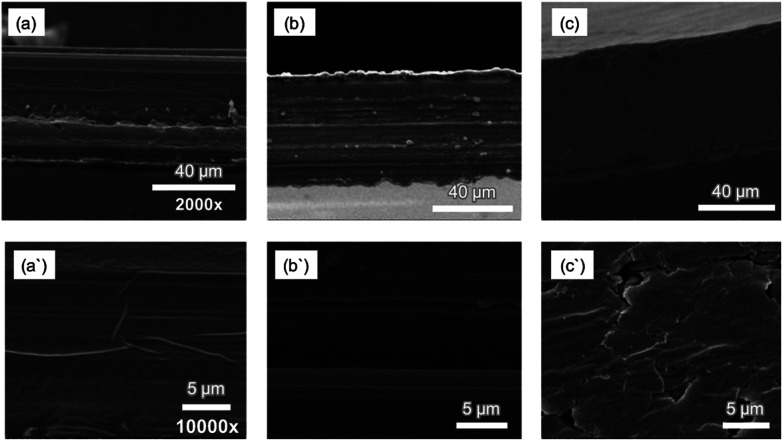
SEM cross section micrographs at two scales: magnification 2000× and 10 000× of CTA-based PIMs with 30 wt% DOP (a and a′); 4 wt% PC-88A, 36 wt% Versatic 10 and 30 wt% DOP (b and b′); and 4 wt% PC-88A and 36 wt% Versatic 10 (c and c′).

The morphology of PIMs with different compositions was investigated using high resolution SEM. Fig. 7 shows SEM cross-sectional images at two scales; magnification 2000× and 10 000× of CTA-based PIMs containing DOP (a and a′); PC-88A, Versatic 10 and DOP (b and b′); and PC-88A and Versatic 10 without DOP (c and c′). All the membranes shown in Fig. 7 were transparent as shown in Fig. 1. The SEM images of CTA-based PIM with only DOP revealed a homogenous and uniform membrane structure (Fig. 7(a′)). The SEM image of the PIM binary system composed of 4 wt% PC-88A and 36 wt% Versatic 10 with 30 wt% DOP as the plasticizer, which offered the best Sc^3+^ ion transport, exhibited a well-oriented multilayer fibrous structure (Fig. 7(b′)). The PIM with the same binary carrier but without DOP (Fig. 7(c′)) is characterized by a non-uniform cracked structure thus illustrating the critical role of the plasticizer in forming a uniform and homogeneous PIM and explaining the poor permeability of this PIM. Consequently, it can be concluded that by adding a plasticizer, both the uniformity and permeability of the membranes increases significantly. Moreover, the PIMs prepared with DOP were thinner and with a low density (compared (b) to (c)) that would further promote high membrane permeability. These results are in agreement with the study of Manzak *et al.*^32^ about the role of the plasticizer in constructing more flexible and softer PIMs and in minimizing the intermolecular interaction forces in their polymer scaffolds thus resulting in higher membrane permeability.

### 3.3 Membrane transport experiments

Membrane transport experiments of metal ions were conducted using PIMs with total binary carrier concentration of 40 wt%, where the ratio between PC-88A and Versatic 10 was varied. When maintaining the percentage of Versatic 10 constant and varying the PC-88A percentage between 1 and 3 wt%, incomplete extraction of Sc^3+^ was observed from a nitrate acid feed solution at pH 4, while more than 6 wt% PC-88A enhanced the transport of the other REM ions studied over that of Sc^3+^ into the 1 M sulfuric acid receiving solution. Therefore, the membrane composition of 40 wt% binary extractant (4 wt% PC-88A + 36 wt% Versatic 10), 40 wt% DOP and 20 wt% CTA, already selected as optimal for the extraction and stripping of Sc^3+^, was found to be optimal for its transport as well.

A PIM with optimal composition mentioned above but without the plasticizer DOP showed very poor permeability for Sc^3+^ in transport experiments.

As described before, the extraction of Sc^3+^ proceeds *via* the exchange of three protons in the binary extractant system.^26^ Membrane transport of the metals is thought to proceed by the following mechanism: the extraction reaction with a cation exchange occurs at the membrane surface of the feed side, followed by the diffusion of the metal species in the membrane from the feed to the receiving side where the metal ion is recovered into the receiving solution.

The driving force in the membrane transport is the concentration gradient of the hydrogen ions between the feed and the receiving solution. As described above, the highly acidic conditions in the receiving solution were crucial for obtaining effective transport across the membrane. The percentage of Sc^3+^ recovery after a 24 h transport was about 36% when a 1 M H_2_SO_4_ receiving solution was used and this value slightly increased with increasing the H_2_SO_4_ concentration to 2 mol L^−1^. However, a decline of the transport efficiency was observed when 3 M H_2_SO_4_ was used as shown in Table 2. This result was contrary to the Le Chatelier's principle according to which with increasing of the concentration gradient of the hydrogen ions the extraction rate should increase accordingly.^33^ However, when the sulfuric acid concentration was higher than 2 mol L^−1^, co-extraction of the acid was observed, which caused a decrease in the pH of the feed solution. When the receiving solution contained 1 M HCl or 1 M HNO_3_, very small amount of Sc^3+^ was recovered after a 24 h operation, as summarized in Table 2. The drop in the pH value in the feed solution to around zero after 6 h of transport was observed, which was due to the fast transport of acids into the feed solution.

**Table 2:** Effect of acid type and concentration in the receiving solution on the transport efficiency for the Sc^3+^ ion and the pH change of the feed solution after 24 h of transport. Remaining experimental conditions as in Fig. 6

Acids	pH^f^		Remaining% of Sc^3+^ in the feed solution	Recovery% of Sc^3+^in the receiving solution
	*t* = 0 h	*t* = 24 h		
1 M H_2_SO_4_	4	3.33	15.7	35.7
2 M H_2_SO_4_	4	3.21	2.5	39.8
3 M H_2_SO_4_	4	3.12	23.5	18.2
1 M HCl	4	<Zero	59.7	12.5
1 M HNO_3_	4	<Zero	68.8	4

**Fig. 8:**
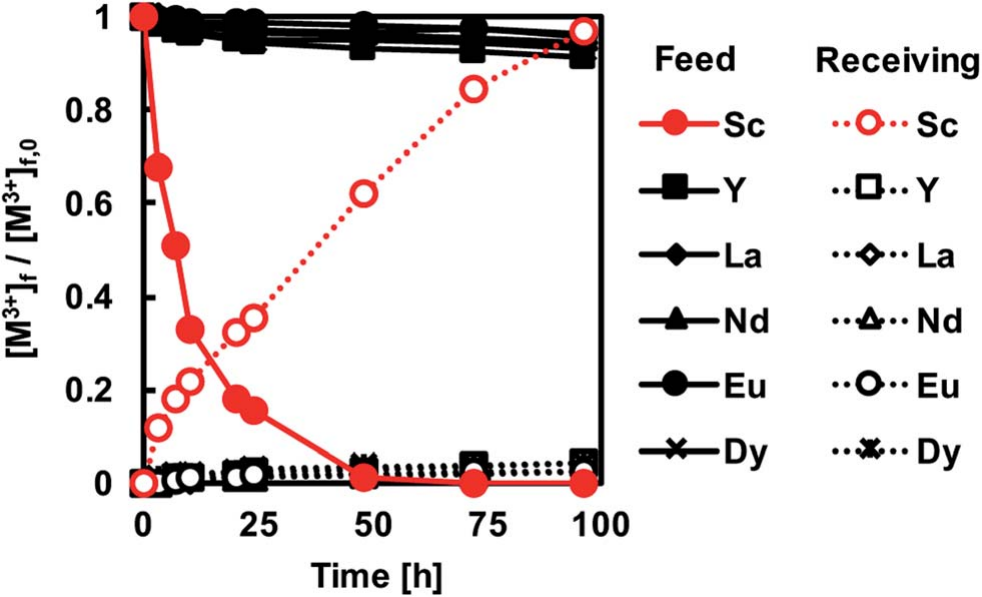
Transport of REM ions across a PIM of optimal composition. Experimental conditions as in Fig. 6.

Fig. 8 shows the transport behaviour of REM ions including Sc^3+^ through the CTA-based PIM with the optimal composition and a receiving solution containing 1 M H_2_SO_4_. It was found that Sc^3+^ concentration in the feed solution decreased relatively fast, while in the receiving solution, the concentration gradually increased. The difference in the rates of depletion and accumulation of Sc^3+^ in both solutions was considered to be the fact that the metal transport was dominated by the diffusion in the membrane. Therefore, the rate of accumulation of the metal ion in the receiving solution was slower in the initial stages of the transport process (Fig. 8). After 96 h from the start of the transport experiment, Sc^3+^ was quantitatively recovered in the receiving solution with a recovery factor of 96.7%, and only a small amount of other REM ions was transported through the membrane. These results demonstrate that the binary carrier membrane containing PC-88A and Versatic 10 is suitable for the selective and quantitative recovery of Sc^3+^ from its nitrate solutions containing other REM ions into a 1 M H_2_SO_4_ receiving solution. The kinetic parameters for the successful transport were calculated and listed in Table 3.

**Table 3:** Kinetic parameters for Sc^3+^ transport. Experimental conditions as in Fig. 8

*K* (h^−1^)	*V* (m^3^)	*A* (m^2^)	[Sc^3+^]^f^_0_ (mol L^−1^)	*P* (m h^−1^)	*J*_0_ (mol m^−2^ s^−1^)
0.0664	5 × 10^−5^	4.9 × 10^−4^	1 × 10^−4^	6.77 × 10^−3^	1.88 × 10^−7^

## 4 Conclusions

This paper reports the development of the first PIM with a binary carrier consisting of PC-88A and Versatic 10 and its use for the selective separation and pre-concentration of Sc^3+^ from nitrate solutions containing other REM ions. The use of the binary carrier allowed easier stripping of Sc^3+^ from the PIM and thus enabled the construction of a successful membrane transport system for Sc^3+^. The transport efficiency of the PIM was found to depend on both the membrane and receiving solution compositions which were optimized as 4 wt% PC-88A + 36 wt% Versatic 10, 40 wt% DOP and 20 wt% CTA for the PIM and 1 M H_2_SO_4_ for the receiving solution. The results obtained in this study illustrate the potential of binary carrier PIMs for the separation and pre-concentration of target chemical species.

## Conflicts of interest

There are no conflicts.

## Supplementary Material
